# Do We Need Surveillance Urethro-Cystoscopy in Patients with Neurogenic Lower Urinary Tract Dysfunction?

**DOI:** 10.1371/journal.pone.0140970

**Published:** 2015-10-29

**Authors:** Ulla Sammer, Matthias Walter, Stephanie C. Knüpfer, Ulrich Mehnert, Beata Bode-Lesniewska, Thomas M. Kessler

**Affiliations:** 1 Neuro-Urology, Spinal Cord Injury Center and Research, University of Zürich, Balgrist University Hospital, Zürich, Switzerland; 2 Institute of Surgical Pathology, University of Zürich, University Hospital Zürich, Zürich, Switzerland; Universitätsmedizin Greifswald, GERMANY

## Abstract

**Purpose:**

To examine the value of surveillance urethro-cystoscopy in patients with neurogenic lower urinary tract dysfunction (NLUTD) in regard to the conflicting literature as it is generally agreed that patients with NLUTD are at increased risk for bladder cancer.

**Materials and Methods:**

In a cross-sectional study, a consecutive series of 129 patients (50 females, 79 males, mean age 51, range 18–88) suffering from NLUTD for at least 5 years was prospectively investigated using urethro-cystoscopy and bladder washing cytology at a single university spinal cord injury (SCI) center.

**Results:**

Due to suspicious urethro-cystoscopy and/or bladder washing cytology findings, 13 (10%) of 129 patients underwent transurethral resection of the bladder lesion and/or random bladder biopsies. Overall, 9 relevant histological findings were found in 5% (7/129) of our patients: bladder melanosis (n = 1), nephrogenic adenoma (n = 3), keratinizing squamous metaplasia (n = 1), intestinal metaplasia (n = 3), and muscle-invasive adenocarcinoma of the bladder (n = 1).

**Conclusions:**

Using surveillance urethro-cystoscopy, we found relevant histological findings in 5% of our patients suffering from NLUTD for at least 5 years. Thus, surveillance urethro-cystoscopy might be warranted, although the ideal starting point and frequency remain to be determined in further prospective studies.

## Introduction

Bladder cancer, as defined by the 10th revision of the International Classification of Diseases (ICD-10) [1], is a common neoplasia in the general population with a recently reported incidence of 151 and a mortality of 52 per 100’000 adults in Europe for the year 2012 [2]. Men are more frequently affected than women with a higher incidence ratio ranging between 1.3 and 6.3 [3]. In men, bladder cancer is the sixth leading cancer worldwide [4]. There are several risk factors associated with bladder cancer, that is, tobacco smoking, increased age, male gender, chronic bladder infection, chronic irritation from indwelling catheters, and exposure to aromatic amines, polycyclic aromatic hydrocarbons and chlorinated hydrocarbons, benzole, cyclophosphamide and radiation [5]. It is generally agreed that patients with neurogenic lower urinary tract dysfunction (NLUTD) due to spinal cord injury (SCI) are at increased risk for bladder cancer [6]. However, there is no consensus on the type and frequency of investigations to detect urological malignancies at an early stage in patients suffering from NLUTD and there is a lack of specific recommendations in the guidelines of the American Urological Association (AUA) and European Association of Urology (EAU) to address this issue. Considering the conflicting literature, we aimed to examine the value of surveillance urethro-cystoscopy in patients with NLUTD due to different neurological disorders including SCI.

## Materials and Methods

### Patients

From January 2011 to November 2013, a consecutive series of 129 patients suffering from NLUTD for at least 5 years was prospectively investigated in a cross-sectional study using urethro-cystoscopy and bladder washing cytology at the SCI Center, University of Zürich, Balgrist University Hospital, Zürich, Switzerland. Study exclusion criteria was current urinary tract infection (UTI), gross hematuria and age <18 years. This study was approved by the local ethics committee, i.e. Kantonale Ethikkommision (KEK) Zürich, Switzerland; study identification number: KEK-ZH-NR: 2010-0207/0 and all patients provided written informed consent according to the Helsinki II declaration.

### Investigations

According to the EAU guidelines, urethro-cystoscopy and bladder washing cytology are both established routine follow-up investigations for patients with NLUTD [5]. At our department, we performed both in addition to a widespread neuro-urological evaluation that includes renal ultrasound and urodynamics. Furthermore, we obtained the medical history, in particular the method of bladder management and the exposure to risk factors for developing bladder cancer such as tobacco smoking, aromatic amines, polycyclic aromatic hydrocarbons, chlorinated hydrocarbons, ionizing radiation, cyclophosphamide, pioglitazone [5].

Patients with a UTI were excluded and adequately treated according to the antibiotic sensitivity pattern before urethro-cystoscopy. For local anesthesia, lidocaine gel (Instillagel^®^ 2x 10 mL, Farco-Pharma GmbH, Germany) was instilled into the urethra and exposed for 10 minutes. Urethro-cystoscopy was performed in a lithotomy or supine position using a rigid or flexible cystoscope in women and men, respectively. After urethro-cystoscopy, we routinely performed a bladder washing cytology. In the case of suspicious urethro-cystoscopy and/or bladder washing cytology findings, the patient was scheduled for transurethral resection of the bladder (TUR-B) and/or random cold biopsies in spinal or general anesthesia. Histological and cytological findings were reported by board certified pathologists and cytologists, respectively, according to findings on routinely processed and stained specimen.

### Outcome Measure

The outcome measures were suspicious urethro-cystoscopy and/or bladder washing cytology findings and relevant histological findings defined as bladder cancer or potentially premalignant lesions.

### Statistical Analysis

Data were normally distributed and are presented as mean and standard deviation (SD). Descriptive statistical analyses were performed using IBM^®^ SPSS^®^ Statistics Version 19.

## Results

Mean age of the 129 patients was 51 ± 16 years (range 18 to 88). 50 (39%) and 79 (61%) were women and men, respectively. The cause of NLUTD was SCI in 89 patients (69%), multiple sclerosis in 20 (16%), myelomeningocele in 8 (6%), cerebral palsy in 4 (3%) and other neurological disorders in 8 (6%). Patients’ characteristics are shown in Table 1.

**Table 1:** Patients’ characteristics.

Neurological disorder	Number of patients (females)	Age at cystoscopy in years*	Duration of neurological disorder in years*	Bladder management		
				I	II	III
Spinal cord injury (69%)	89 (25)	50±15 (18–88)	15±9 (5–45)	29	43	17 (10/7)
Multiple sclerosis (16%)	20 (16)	55±11 (36–73)	22±10 (6–47)	7	5	8 (8/0)
Myelomeningocele (6%)	8 (3)	30±19 (21–75)	30±19 (21–75)	3	5	0
Cerebral palsy (3%)	4 (3)	56±8 (45–65)	56±8 (45–65)	3	0	1 (1/0)
Other (6%)	8 (3)	70±12 (49–88)	19±25 (5–80)	5	0	3 (3/0)
All	129 (50)	51±16 (18–88)	19±14 (5–80)	47	53	29 (22/7)

Bladder management: I = Spontaneous voiding, II = Aseptic intermittent self-catheterization and III = Indwelling catheter (suprapubic/transurethral).

* Data presentation: mean ± standard deviation (range).

**Fig 1:**
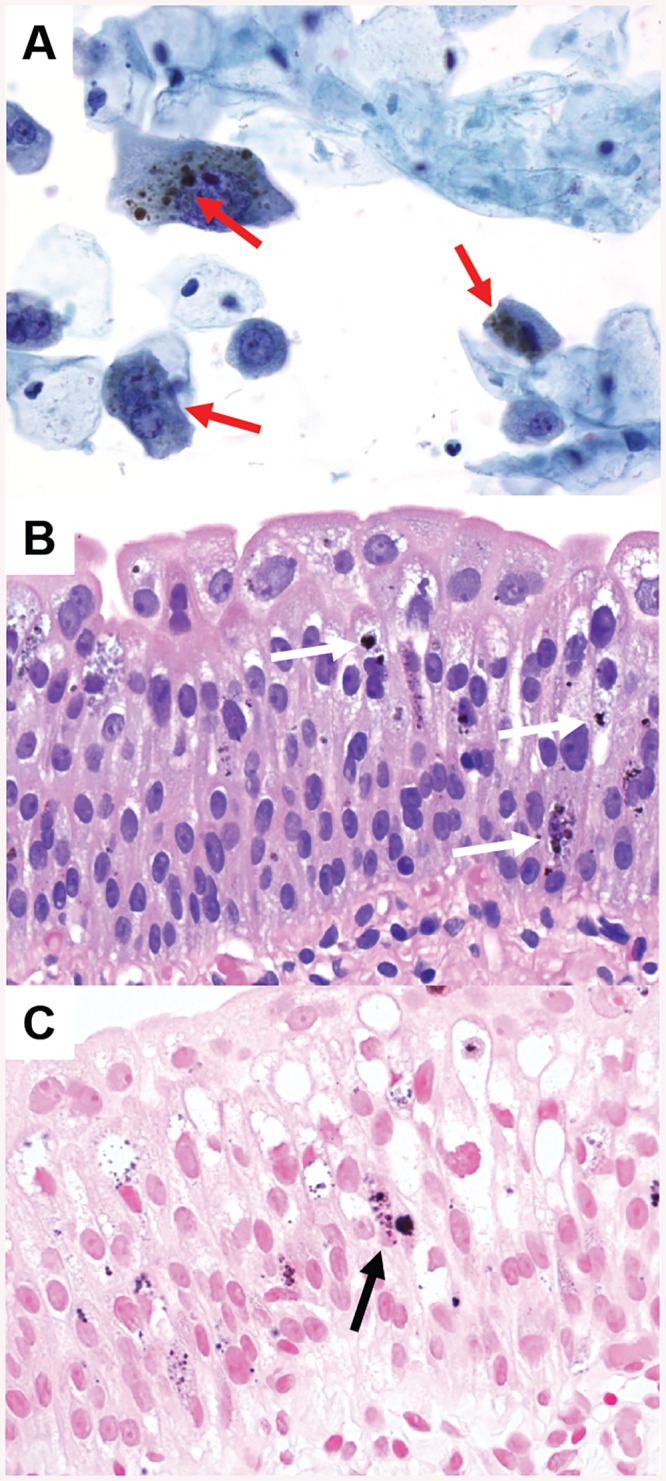
Melanosis of the urothelium in a 56 years old woman (Table 3, Patient 12). **A**—Bladder washing with dark brown and black pigment in urothelial cells (arrows) (Papanicolaou stain; original magnification 400x). **B—**Bladder mucosa biopsy containing dark brown and black pigment in urothelial cells of all levels (arrows) (Hematoxylin and eosin stain; original magnification 400x). **C—**Negativity of the intraepithelial pigment in an iron stain (arrow) excluding hemosiderin pigment deposits (Iron stain; original magnification 400x).

**Fig 2:**
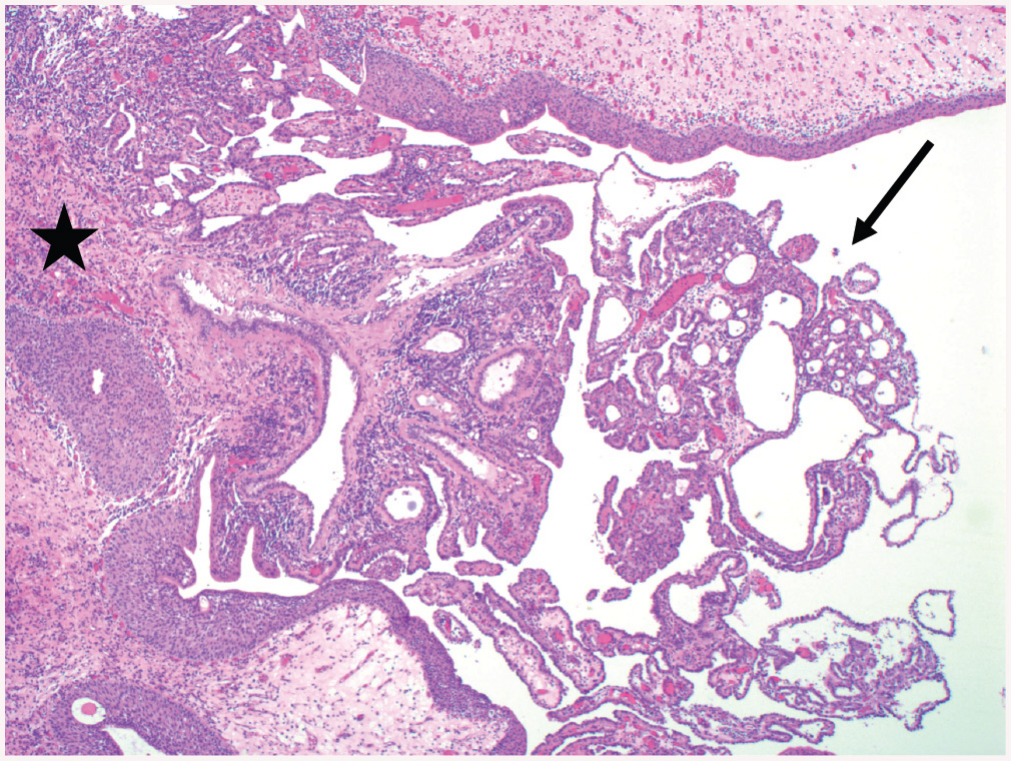
Nephrogenic adenoma in a 21 years old woman (Table 3, Patient 10). Intraluminal polypoid mass (arrow), covered by cylindrical non-dysplastic epithelium and containing bland glands, accompanied by some inflammatory cells. The bladder mucosa at the base of the lesion (asterisk) shows chronic inflammation (Hematoxylin and eosin stain; original magnification 25x).

**Fig 3:**
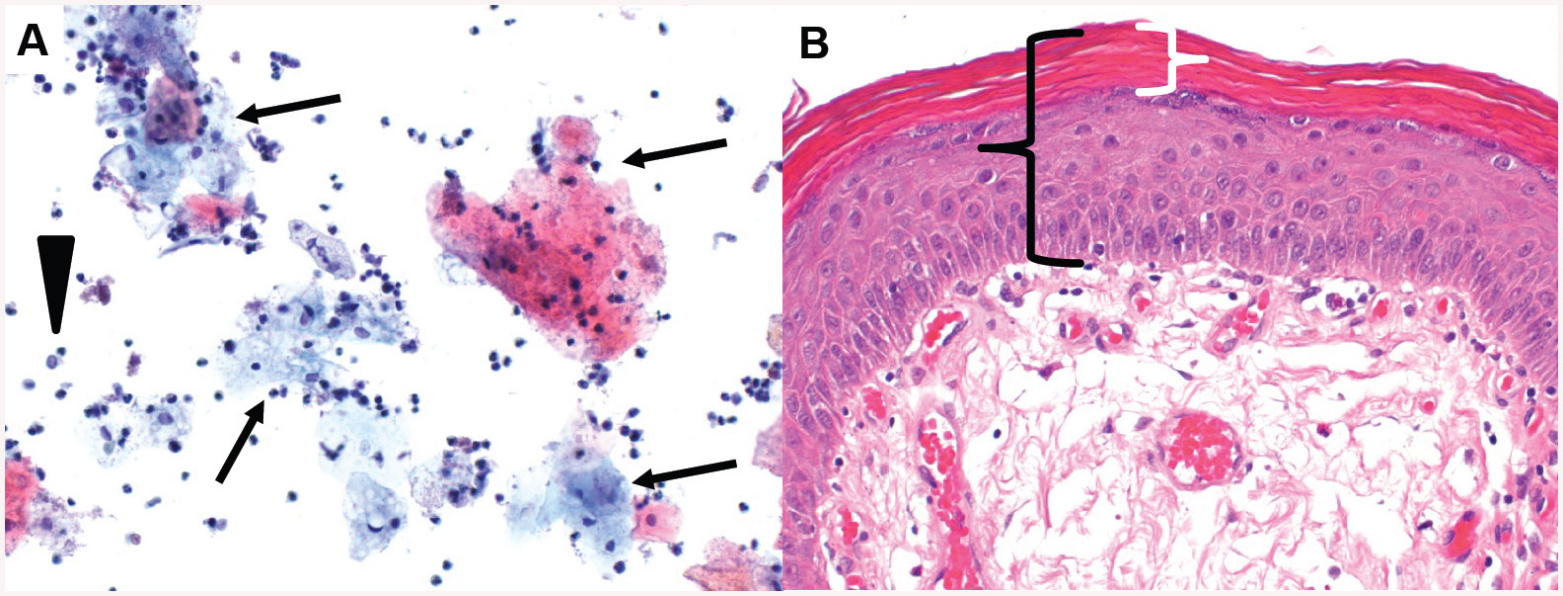
Extensive keratinizing squamous metaplasia of the urothelium in a 43 years old man (Table 3, Patient 7). **A**—Bladder washing with numerous, often anucleated squamous cells (arrows). Only rare, degenerated urothelial cells (arrowhead) and many neutrophils in the background (Papanicolaou stain; original magnification 200x). **B—**On biopsy, bladder mucosa is covered by non-dysplastic squamous epithelium (black bracket) with a thick anucleated keratinized superficial layer (white bracket) (Hematoxylin and eosin stain; original magnification 200x).

**Fig 4:**
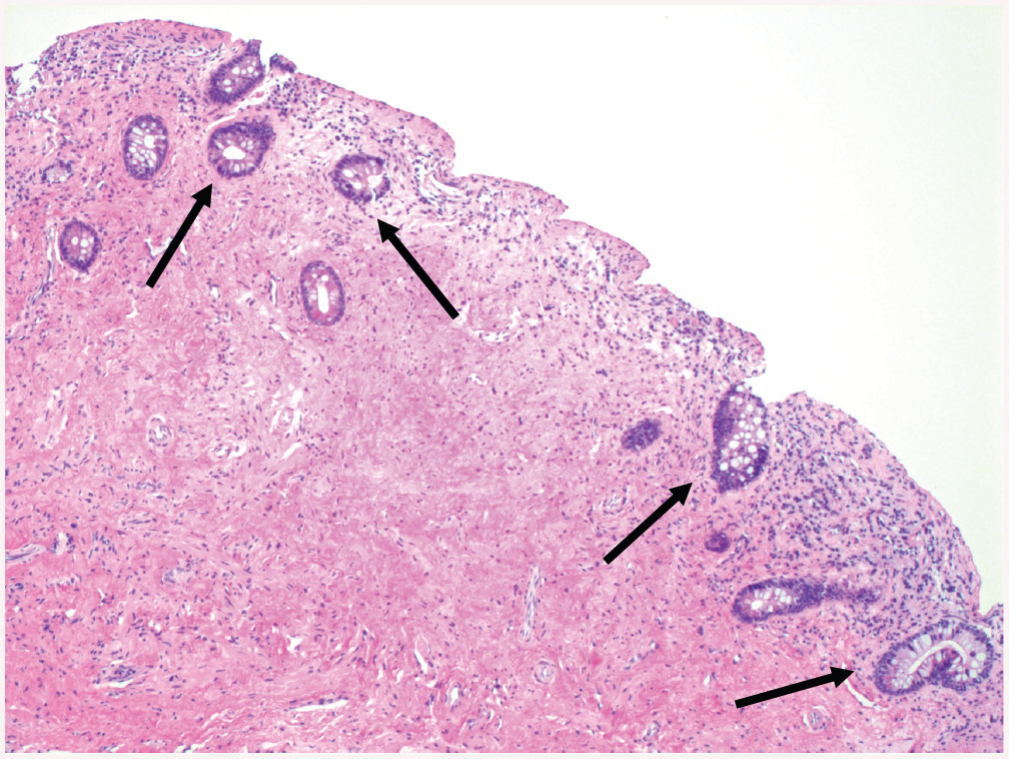
Intestinal metaplasia in a 45 years old woman (Table 3, Patient 11). Intestinal type glands in the bladder biopsy (arrows). Denuded surface of the mucosa containing some inflammatory cells (Hematoxylin and eosin stain; original magnification 50x).

**Fig 5:**
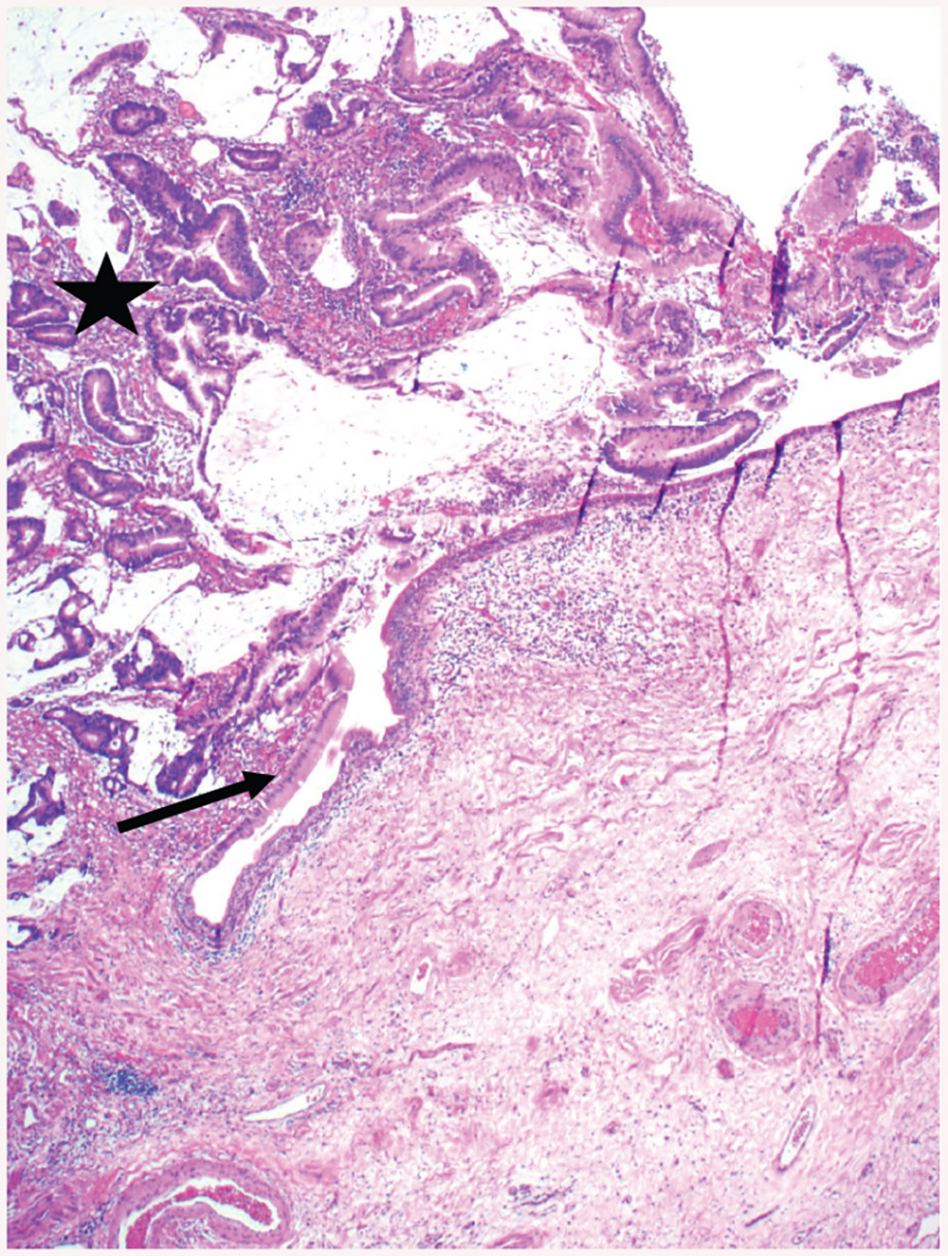
Mucinous adenocarcinoma in a 59 years old man (Table 3, Patient 8). Intraluminal polypoid mass (asterisk) consisting of malignant glands with intra- and extracellular mucus and infiltrating growth. The arrow points to the transition zone between urinary type epithelium and cylinder cells of the intestinal metaplasia (Hematoxylin and eosin stain; original magnification 25x).

Overall, 58 (45%) of the 129 patients were exposed to at least one risk factor for bladder cancer (Table 2). Due to suspicious urethro-cystoscopy and/or bladder washing cytology findings, 13 (10%) of the 129 patients underwent transurethral resection of the bladder lesion and/or random bladder biopsies (Table 3). In total, 9 relevant histological findings were found in 5% (7/129) of our patients: Bladder melanosis (n = 1, Fig 1), nephrogenic adenoma (n = 3, Fig 2), keratinizing squamous metaplasia (n = 1, Fig 3), intestinal metaplasia (n = 3, Fig 4) and muscle-invasive adenocarcinoma of the bladder (n = 1, Fig 5). The patient with bladder cancer underwent bilateral pelvic lymphadenectomy and radical cystoprostatovesiculectomy with a continent catheterizable reservoir and final histology revealed pT3b pN0 (0/15) cM0 G3 mucus-producing adenocarcinoma of the bladder.

**Table 2:** Patients’ exposure to risk factors for bladder cancer.

Neurological disorder	Exposure to risk factors*	Tobacco	Aromatic amines	Polycyclic aromatic hydrocarbons	Chlorinated hydrocarbons	Ionizing radiation
Spinal cord injury (n = 89)	45 (51%)	40	12	5	3	3
Multiple sclerosis (n = 20)	5 (25%)	5	0	0	0	0
Myelomeningocele (n = 8)	3 (38%)	1	1	1	0	0
Cerebral palsy (n = 4)	1 (25%)	1	0	0	0	0
Other (n = 8)	4 (50%)	3	1	0	0	2
All (n = 129)	58 (45%)	50	14	6	3	5

* Patients were exposed to at least one risk factor (multiple exposures possible).

**Table 3:** Characteristics of the patients undergoing transurethral resection of the bladder lesion and/or random bladder biopsies.

No.	Gender	Age	Neurological disorder	Injury level	AIS	Duration of neurological disorder	Bladder management	Cystoscopy	Cytology	Histopathology	History of tobacco consumption (pack years)	Exposure to other risk factors
1	Male	59	SCI	T5	A	5	II	Suspicious reddish bladder lesions	Squamous metaplasia	Follicular cystitis	No	No
2	Male	26	SCI	T5	A	7	II	Suspicious reddish bladder lesions	Squamous metaplasia	Follicular cystitis	Yes (7)	No
3	Female	45	SCI	T10	D	9	I	Exophytic bladder tumor	Squamous metaplasia	Papillary inflammatory lesions	Yes (39)	No
4	Male	69	SCI	C4	D	14	II	Suspicious reddish bladder lesions	Squamous metaplasia	Nonkeratinizing squamous metaplasia & follicular cystitis	Yes (30)	Yes^1^
5	Male	43	SCI	C5	C	16	III (transurethral)	Exophytic bladder tumor	Inflammatory cells	Eosinophilic cystitis	Yes (20)	Yes^1^
6	Male	52	SCI	C8	A	20	II	Exophytic bladder tumor	Inflammatory cells	Nephrogenic adenoma & cystitis cystica	No	No
7	Male	43	SCI	T6	A	24	II	Suspicious reddish bladder lesions	Squamous metaplasia	Keratinizing squamous metaplasia	Yes (3)	Yes^1^,^2^
8	Male	59	SCI	T5	A	33	II	Exophytic bladder tumor	Squamous metaplasia	Muscle-invasive adenocarcinoma of the bladder & intestinal metaplasia	No	No
9	Female	62	MS	-	-	30	III (suprapubic)	Exophytic bladder tumor	Inflammatory cells	Nephrogenic adenoma, nonkeratinizing squamous metaplasia & intestinal metaplasia	No	No
10	Female	21	MMC	-	-	21	II	Exophytic bladder tumor	Inflammatory cells	Nephrogenic adenoma	No	No
11	Female	45	CP	-	-	45	III (suprapubic)	Exophytic bladder tumor	Inflammatory cells	Intestinal metaplasia & cystitis cystica et glandularis	No	No
12	Female	56	CP	-	-	56	I	Multiple black bladder spots	Melanosis	Melanosis of the bladder	No	No
13	Male	88	Other (GBS)	-	-	10	III (suprapubic)	Exophytic bladder tumor	low grade urothelial cell atypia	Chronic cystitis	No	No

Bladder management: I = Spontaneous voiding, II = Aseptic intermittent self-catheterization and III = Indwelling catheter (suprapubic/transurethral).

AIS = American Spinal Injury Association Impairment Scale, CP = Cerebral palsy, GBS = Guillain-Barré syndrome,

MMC = Myelomeningocele, MS = Multiple sclerosis and SCI = Spinal cord injury.

^1^ Exposure to aromatic amines

^2^ Exposure to polycyclic aromatic hydrocarbons.

## Discussion

Using surveillance urethro-cystoscopy, we detected relevant histological findings in 5% of our patients suffering from NLUTD for at least 5 years. Considering this important percentage of clinically relevant findings, surveillance urethro-cystoscopy might be warranted, although the ideal patient population, starting point and frequency remain to be determined in further prospective studies.

In comparison to the general population, an up to 25 times higher risk of developing bladder cancer has been reported in patients with SCI relying on chronic indwelling catheters [7]. This method of bladder management leads to chronic irritation of the bladder mucosa [8] and chronic urinary tract infections (UTI) [6] while serving as an independent risk factor for bladder cancer in SCI patients [7]. Bladder cancer has been reported to be present in SCI patients at a lower age and a higher stage, when diagnosed [6], compared to the general population.

Gross hematuria, a well-known phenomenon in SCI patients with indwelling catheters, has a significantly higher incidence the longer the duration of catheterization is [9]. The initiation of a routine screening protocol might be useful with the objective of minimizing morbidity and mortality of bladder cancer in patients suffering from NLUTD, since typical clinical symptoms of bladder cancer such as gross hematuria may be absent [9]. Although the literature is limited, microscopic hematuria has been reported to be present in 15% of SCI patients [10]. Yet, the specificity of urine dipstick is restricted as microscopic hematuria may result from various reasons, that is calculi, infections or use of certain drugs [11]. Therefore, the interpretation of microscopic hematuria is challenging in this population and needs a patient-tailored approach.

With no consensus on how to manage patients with NLUTD to detect urological malignancies at an early stage, the need for establishing suitable diagnostic methods is substantial. Ultrasound of the bladder, a non-invasive diagnostic tool, is reported to be inferior to urethro-cystoscopy in identifying bladder cancer in patients with hematuria [12] and therefore inappropriate to replace urethro-cystoscopy and bladder washing cytology. However, the role of urethro-cystoscopy and bladder washing cytology for surveillance has not yet been clearly determined and a cost-benefit analysis is still up for discussion regarding this challenging patient population.

According to the consortium for spinal cord medicine [13], SCI patients should undergo an extensive annual urological evaluation program including urethro-cystoscopic investigation in addition to urodynamics to achieve a sufficient functional/anatomical assessment of the lower urinary tract system. Navon et al. recommended the performance of annual urethro-cystoscopy in SCI patients beginning 10 years after injury and in patients with recurrent or chronic UTI resulting in the diagnosis of squamous cell bladder cancer at early stages with consecutive increase of long-term survival [14]. Annual urethro-cystoscopy and urine cytology with routine random biopsies every 1 to 2 years in individuals with SCI was advocated by Broecker et al. [10]. In contrast, Hamid et al. did not recommended routine surveillance urethro-cystoscopy and biopsy in patients with a neuropathic bladder and chronic suprapubic indwelling catheters due to high screening costs with low detection rate and associated morbidity [8]. Importantly, there are no generally agreed recommendations regarding follow-up evaluation of the neuro-urological patient and there is a complete lack of high-evidence level studies on this topic [15].

In an attempt to minimize morbidity caused by urethro-cystoscopy, antibiotic prophylaxis might be considered in high-risk patients suffering from NLUTD in order to prevent symptomatic UTI. In patients with SCI being at risk of the development of autonomic dysreflexia due to bladder distension during urethro-cystoscopy might profit from using a less traumatic flexible instead of a rigid cystoscope while being on continuous cardiovascular monitoring at the same time, that is, blood pressure and heart rate [16]. To improve the detection rate of bladder cancer, additional diagnostic tools might be useful. Therefore, we routinely perform bladder washing cytology during urethro-cystoscopy as Stonehill et al. reported good sensitivity (71%) and high specificity (97%) for diagnosis of bladder cancer in SCI patients [17]. As unspecific multifocal flat lesions of the bladder mucosa can often be found by urethro-cystoscopy in patients suffering from NLUTD, particularly in patients with chronic indwelling catheter due to chronic irritation/inflammation, bladder-washing cytology might be a good indicator for the presence of carcinoma in situ (CIS) leading to subsequent biopsy and treatment. Nevertheless, the limitation of cytology concerning the high rate of false negative results in detecting low-grade tumors [17] must be taken into consideration.

In the present study using surveillance urethro-cystoscopy, relevant histological findings varied widely and included bladder melanosis, nephrogenic adenoma, keratinizing squamous metaplasia, intestinal metaplasia, and mucus-producing adenocarcinoma. Considering the fact that relevant histological findings were present even in 15% (2/13) of the patients with NLUTD who voided spontaneously, NLUTD itself might be a risk factor for the development of bladder cancer as suggested by Kalisvaart et al. [18].

Although a relevant high percentage (45%) of patients was exposed to at least one risk factor for bladder cancer, we did not find any dysplastic lesions. Bladder melanosis (Fig 1), a rare entity with less than 20 cases reported up to date, is characterized by abnormal/excessive deposits of melanin in the urothelium without any signs of atypia [19]. In general, bladder melanosis is considered to be a benign lesion but it has been described as a potential premalignant factor associated with primary melanoma of the bladder [20], high grade transitional cell carcinoma of the bladder [21] and the upper urinary tract [19]. Although our patient with bladder melanosis suffered from NLUTD due to cerebral palsy, it is unknown, if there is a causal relationship between NLUTD and the development of bladder melanosis or if it is just a coincidental histological finding. Nephrogenic adenoma (Fig 2) is an uncommon metaplastic lesion of the urothelium found in all locations of the urinary tract often with multifocal appearance and most commonly seen in the urinary bladder [22, 23]. Etiology and biological potential are still unclear, but it seems to be attributed to urothelial irritation by trauma, previous surgery, urinary calculi, radiation, chronic infections and urinary catheterization [22, 23]. Clinical symptoms, when present, are non-specific such as lower urinary tract symptoms and hematuria [22, 23]. Therapeutic options vary from watchful waiting to cystectomy [23], whereas in most cases a transurethral resection is favored [24], as it was the case in our 3 patients with a nephrogenic adenoma. A high recurrence rate up to 80% has been reported [23]. Although nephrogenic adenoma is usually thought to be a benign entity [22], there might be some potential for malignant transformation as its occurrence has been reported in association with transitional cell carcinoma [23] and moderately differentiated adenocarcinoma of the bladder in a patient with NLUTD [24]. Because of the high recurrence rate of the nephrogenic adenoma and its assumed potential for neoplastic transformation, regular follow-up should be considered. Keratinizing squamous metaplasia (Fig 3) is rare and characterized by a replacement of the urothelial layer with stratified keratinizing squamous epithelium due to chronic inflammation/irritation of the bladder mucosa caused by different predisposing factors such as urinary tract infections, indwelling catheters, fistula, urinary calculi, bladder outlet obstruction, bladder extrophy and neurogenic bladder [25]. Keratinizing squamous metaplasia of the bladder is postulated to be a pre-malignant condition leading to a higher risk of the development of bladder cancer, especially if dysplasia and/or extensive keratinization is present [26]. Most commonly, keratinizing squamous metaplasia of the bladder is associated with co-existing or subsequent squamous cell carcinoma and transitional cell carcinoma [27]. The risk of developing bladder cancer [26] in patients with keratinizing squamous metaplasia of the bladder is high as progression has been reported in 27% with a variable period of latency between 4 and 28 years [27]. In keratinizing squamous metaplasia of the bladder, a complete transurethral resection is considered to be the therapy of choice [26] and a close follow-up using urethro-cystoscopy and bladder washing cytology is highly advocated in order to detect malignant transformation at early stages [27].

Intestinal metaplasia (Fig 4) of the bladder is characterized by the presence of intestinal-type epithelium and/or goblet cells in the bladder urothelium and is suggested to occur as a result of chronic inflammation and irritating stimuli such as urinary tract infections, calculi, neurogenic bladder, bladder extrophy, and long-term catheterization [25, 28]. Clinical symptoms reported by patients contain hematuria, dysuria, urgency and mucusuria as a sign of mucus production/secretion of these lesions [25, 28]. Intestinal metaplasia of the bladder is often associated with adenocarcinoma of the bladder [28] implying pre-malignant characteristics [25] comparable to Barrett’s metaplasia of the esophagus [29]. Thus, considering the potential of intestinal metaplasia to progress to a malignant condition such as adenocarcinoma of the bladder, we regularly perform urethro-cystoscopy and bladder washing cytology in such patients to ensure an early detection of malignant transformation. Importantly, patients with SCI have a higher risk to develop bladder cancer compared to the general population [18]. We agree with the existing literature [6], that bladder cancer appears to be a late complication in patients suffering from NLUTD due to SCI, i.e. bladder cancer was detected in our patient 33 years post-injury.

In patients with muscle-invasive bladder cancer, bilateral pelvic lymphadenectomy and radical cystectomy combined with urinary diversion is mandatory as in our SCI patient with co-existing intestinal metaplasia and mucus-producing adenocarcinoma of the bladder (Fig 5).

Patients with NLUTD require an extensive and specific work-up before embarking on an individualized therapy taking into account the patients’ medical and physical condition as well as their expectations [30]. Considering the high incidence of relevant histological findings in the present study, surveillance urethro-cystoscopy and bladder washing cytology might be warranted. While awaiting the results of well-designed risk-stratification studies, we recommend performing surveillance urethro-cystoscopy and bladder washing cytology on patients suffering from NLUTD for at least 5 years on a regular short-term basis, that is, every 1 to 2 years. This has also been suggested in a very recent retrospective study of surveillance urethro-cystoscopy in SCI patients relying on an indwelling urethral or suprapubic catheter [31]. Although our recommendation is not yet based on high-evidence level studies, this policy seems justified considering the highly relevant, potentially life threatening consequences of not detecting and consequently not treating a pre-malignant and/or malignant lesion.

Metaplastic lesions such as nephrogenic adenoma, keratinizing squamous metaplasia and intestinal metaplasia may progress to a malignant condition but the underlying mechanisms involved are unclear and remain to be elucidated. Given the heterogeneous nature and management of neurogenic LUTD, it would be of great interest to know which sub-group of patients is at highest risk to develop relevant histological lesions. In addition, further studies are needed to determine the ideal starting point and frequency of surveillance urethro-cystoscopy. Thus, prospective large-scale multicenter studies are highly warranted to investigate these still unanswered questions and to further improve the management of patients with NLUTD.

### Study Limitations

The present study has some limitations. Although we evaluated a well-defined patient population with NLUTD, there are limitations that should be addressed. Most of our patients suffered from SCI, that is, patients with other neurological disorders were under-represented. Our unit is part of a highly specialized university SCI center so that a negative selection bias, that is, inclusion of more severe cases, cannot to be completely ruled out. Considering that we only studied patients suffering from NLUTD for at least 5 years, it is unclear whether the results could be extrapolated to patients with a shorter exposure time to the neurological disease. Due to the cross-sectional study design, randomization was not possible and patients with versus without surveillance urethro-cystoscopy could not be compared. Moreover, because of the limited patient number in some groups, we did not perform sub-group analysis. Nevertheless, the present study was prospective and representative of daily clinical practice.

## Conclusions

In patients suffering from NLUTD for at least 5 years, we detected suspicious urethro-cystoscopy and/or bladder washing cytology findings in 10% and relevant histological findings in 5%. The relevant histological findings varied widely, that is, from bladder melanosis to muscle-invasive mucus-producing adenocarcinoma of the bladder. Considering the important percentage of clinically relevant findings, surveillance urethro-cystoscopy might be warranted. However, we cannot conclude from our data which sub-group of patients with NLUTD would profit most from surveillance urethro-cystoscopy. In addition, starting point and frequency of surveillance urethro-cystoscopy remain unclear. Thus, further well-designed, adequately powered and sampled prospective studies are needed to investigate these highly relevant issues.

## Supporting Information

S1 Dataset De-identified patients’ data used in this study. (XLSX)

S1 Fig STROBE checklist part 1. (PDF)

S2 Fig STROBE checklist part 2. (PDF)
