# Supplementary material for: Do We Need Surveillance Urethro-Cystoscopy in Patients with Neurogenic Lower Urinary Tract Dysfunction?
Source: PLoS One. 2015 Oct 29;10(10):e0140970. doi: 10.1371/journal.pone.0140970 (PMC4626398; doi:10.1371/journal.pone.0140970)
Supplement: S2 Fig — (PDF) [file pone.0140970.s003.pdf]

**Discussion**		
Key results	18	Summarise key results with reference to study objectives ✓
Limitations	19	Discuss limitations of the study, taking into account sources of potential bias or imprecision. Discuss both direction and magnitude of any potential bias ✓
Interpretation	20	Give a cautious overall interpretation of results considering objectives, limitations, multiplicity of analyses, results from similar studies, and other relevant evidence ✓
Generalisability	21	Discuss the generalisability (external validity) of the study results ✓
**Other information**		
Funding	22	Give the source of funding and the role of the funders for the present study and, if applicable, for the original study on which the present article is based ✓

*Give information separately for exposed and unexposed groups.

Note: An Explanation and Elaboration article discusses each checklist item and gives methodological background and published examples of transparent reporting. The STROBE checklist is best used in conjunction with this article (freely available on the Web sites of PLoS Medicine at http://www.plosmedicine.org/, Annals of Internal Medicine at http://www.annals.org/, and Epidemiology at http://www.epidem.com/). Information on the STROBE Initiative is available at www.strobe-statement.org.

Zürich, Dec 18th 2014

Matthias Walter
